# Attentional and affective consequences of technology supported mindfulness training: a randomised, active control, efficacy trial

**DOI:** 10.1186/s40359-016-0168-6

**Published:** 2016-11-29

**Authors:** Sheffy Bhayee, Patricia Tomaszewski, Daniel H. Lee, Graeme Moffat, Lou Pino, Sylvain Moreno, Norman A. S. Farb

**Affiliations:** 1 Department of Psychology, University of Toronto Mississauga, 3359 Mississauga Rd., Mississauga, ON L5L 1C6 Canada; 2 University of Colorado Boulder, Boulder, CO USA; 3 Interaxon Inc., Toronto, Canada; 4 Simon Fraser University, Burnaby, Canada

## Abstract

**Background:**

Mindfulness training (MT) programs represent an approach to attention training with well-validated mental health benefits. However, research supporting MT efficacy is based predominantly on weekly-meeting, facilitator-led, group-intervention formats. It is unknown whether participants might benefit from neurofeedback-assisted, technology-supported MT (N-tsMT), in which meditation is delivered individually, without the need for a facilitator, travel to a training site, or the presence of a supportive group environment. Mirroring the validation of group MT interventions, the first step in addressing this question requires identifying whether N-tsMT promotes measurable benefits. Here, we report on an initial investigation of a commercial N-tsMT system.

**Methods:**

In a randomized, active control trial, community-dwelling healthy adult participants carried out 6 weeks of daily practice, receiving either N-tsMT (*n* = 13), or a control condition of daily online math training (*n* = 13). Training effects were assessed on target measures of attention and well-being. Participants also completed daily post-training surveys assessing effects on mood, body awareness, calm, effort, and stress.

**Results:**

Analysis revealed training effects specific to N-tsMT, with attentional improvements in overall reaction time on a Stroop task, and well-being improvements via reduced somatic symptoms on the Brief Symptom Inventory. Attention and well-being improvements were correlated, and effects were greatest for the most neurotic participants. However, secondary, exploratory measures of attention and well-being did not show training-specific effects. N-tsMT was associated with greater body awareness and calm, and initially greater effort that later converged with effort in the control condition.

**Conclusions:**

Preliminary findings indicate that N-tsMT promotes modest benefits for attention and subjective well-being in a healthy community sample relative to an active control condition. However, the findings would benefit from replication in a larger sample, and more intensive practice or more comprehensive MT instruction might be required to promote the broader benefits typically reported in group format, facilitated MT.

**Trial registration:**

Current Controlled Trials ISRCTN43629398. Retrospectively registered on June 16, 2016.

**Electronic supplementary material:**

The online version of this article (doi:10.1186/s40359-016-0168-6) contains supplementary material, which is available to authorized users.

## Background

Modern mindfulness training (MT) aims to apply ancient contemplative traditions to reduce human suffering. The most well-studied MT programs represent clinically-efficacious appropriations of these traditions [1], interventions increasingly recognized for their ability to reduce stress, improve emotion regulation, and strengthen attentional control [2–4]. MT involves changing how one relates to life experience, a transformation initiated by intentionally directing attention away from conceptual thought towards physical sensations with an attitude of curiosity, acceptance and kindness [5]. During formal meditation practice, distractions inevitably arise; the meditator is taught to acknowledge intrusions and non-judgmentally return attention to the breath [6], thus supporting a relaxed but attentive awareness, a ‘decentered’, reflective relationship with thoughts, feelings, and sensations [7]. This reflective stance stands in contrast to seemingly obligatory habits of avoiding or pursuing experiences that are thought to lie at the heart of many modern affective disorders [8, 9].

One criticism of the growing MT literature is that there is little evidence defining the ‘minimum dose’ for successful training. MT is most widely studied via manualized, multifaceted clinical interventions, which prescribe an hour or more of daily practice over 8 weeks, combined with weekly-meetings in a facilitator-led group course format [10, 11]. This dose and duration is largely a product of historical precedent rather than evidence-based medicine. Indeed, the MT ‘minimal dose’ may be substantially smaller than the status quo: group interventions as brief as 20 min a day for 4 days appear to produce cognitive, affective and physiological benefits [12, 13]. A similar lack of evidence surrounds the use of group rather than individual interventions. Individualized, technology-supported MT (tsMT) approaches offer compelling advantages of customizing training to participant needs, addressing concerns around time commitment, and reaching interested practitioners who do not have access to group-facilitated MT programs. Investigating the possibility of efficacious, individualized, tsMT is therefore of significance for extending MT’s benefits to a larger population.

In exploring any new therapeutic intervention, clinical trials often advance from concerns around safe dose (Phase I), uncontrolled efficacy (Phase II), to larger, actively controlled designs (Phases III and IV) [14]. In the case of tsMT, tens of thousands of users already employ this technology without evidence for elevated risk of adverse events. Yet despite its rapid popularization, there are also few (if any) experimental demonstrations of tsMT efficacy. Thus an appropriate first step in beginning MT research may be the exploration of whether tsMT has therapeutic efficacy. Given the inevitable expectancy effects involved in using therapeutic technology, some degree of experimental control seems necessary to the investigation. The current study was designed to address this research gap, i.e., to explore whether tsMT has therapeutic efficacy at its most common dose, relative to an active control training condition.

### Targets of mindfulness training

In assessing MT efficacy, the areas of *attention* and *subjective well-being* are the most well-established proximal targets of change. In contemplative theory, the cultivation of attentional control allows practitioners to observe emotional experiences without obsession or avoidance, yielding benefits to well-being, including, but not limited to the promotion of a relaxation response [15, 16]. This account is consistent with modern psychological theory, in which negative health consequences are associated with both habitual rumination [17] and suppression of emotional experience [18]. Accordingly, the extent to which intensive meditators are able to cultivate attentional control has been associated with improvements in self-reported adaptive socioemotional functioning [19]. While the dynamic interplay between attention and well-being warrants further investigation, one might reasonably expect MT-related improvements in attention and well-being to be correlated in magnitude.

Distinct studies support the idea that attention and well-being are cultivated through MT. Attention appears to be consistently impacted by MT [20–22], with effects most pronounced after intensive training. For example, 3-months of intensive MT improved the ability to sustain attention during a dichotic listening task as evidenced by faster reaction times in response to a deviant tone, and reduced attentional blink responses when compared to controls [23, 24]. Experienced meditators have shown elevated performance on classic tests of attention such as the Stroop task and the D2 Concentration and Endurance task [25]. Additionally, long-term meditation practice has been found to reduce attentional blink in older adults when compared to age-matched and younger adults [26]. In neural terms, extensive MT appears to increase activation in executive attention networks [27], changes which may correlate with behavioral improvements in sustained attention and error monitoring [28]. It is unknown whether these benefits begin to manifest after shorter courses of attention training, although attention likely improves gradually with training.

Complementing findings of improved attention, MT has been consistently associated with improved subjective well-being. Mindfulness-based Stress Reduction (MBSR) and related programs have been found to improve mood and self-reported emotional health [29], and are associated with improvements in immune system functioning [30], stress [31], and emotion regulation [32]. MT is predicated on teaching participants to respond non-judgmentally rather than reacting out of habit to stressful events by focusing on dynamic sensory stimuli, such as the breath, body, or sounds and sensations of eating and walking. As participants learn these skills, top-down control processes are thought to regulate affective appraisals that lead to a reduction in stress responses [33]. Neurally, MBSR-related improvements in well-being have been associated with less suppression of interoceptive processing following emotional stress, as indexed by reduced stress-related suppression of the right posterior insula [34], the putative primary representation cortex for feeling states within the body [35]. In this study, less insula suppression was linked to lower severity of depressive symptoms in a community sample. Taken together, the effects of relatively brief, tsMT interventions can be assessed using well-established metrics of attention and subjective well-being.

### Technology-supported mindfulness training

Despite tsMT’s promise of expanded access and training customization, several challenges are apparent in translating the training from manualised, group-led MT interventions. The technology must address several important elements of more conventional MT, such as providing a motivating training experience, and useful feedback to normalize and direct training efforts. Neurofeedback is one promising method avenue for tsMT, in which some aspect of brain activity is reported back to participants in real-time. Neurofeedback-assisted tsMT (N-tsMT) has the potential for motivating practice by providing brain activity readings that would normally be inaccessible to the practitioner, and these signals may cultivate an expectation of customized training that would be absent in tsMT applications that rely on pre-recorded lessons and guided meditations. While several neurofeedback modalities exist [36], only electroencephalography has already been featured in commercial applications. We focus here on EEG-based N-tsMT, which involves training to modulate brain activity in response to non-invasive measurement of scalp electrical potentials along one or more electrical frequency bands.

While it is likely that particular neurofeedback algorithms have greater efficacy than others for training cultivating particular forms of attention or well-being, comparing algorithms may be premature when investigating whether N-tsMT can promote cognitive and affective benefits. A variety of neurofeedback algorithms have been employed in laboratory settings [37–42], with comparable benefits across a variety of cognitive domains, including sustained attention, executive function, memory, spatial rotation, complex psychomotor skills, reaction time, intelligence, mood, and well-being [43]. Similarly, several distinct lab-based neurofeedback algorithms for meditation have been linked to greater subjective well-being [44, 45]. There is presently no consensus on the optimal algorithm for computing N-tsMT feedback, and as most studies have not used active control comparisons, it is unclear that any neurofeedback algorithm promotes the many benefits linked to N-tsMT practice.

Given a lack of agreement on an optimal neurofeedback algorithm from lab-based studies, and the current availability of a commercial N-tsMT platforms, it may be prudent to first investigate whether existing N-tsMT applications are beneficial before investigating particular training mechanisms or comparing feedback algorithms. After all, if there is no significant benefit to attention or well-being, then arguments over algorithm efficacy are irrelevant. Furthermore, the MT instruction rather than the presence of neurofeedback may be the critical mechanistic ingredient- any paradigm that promotes motivation to engage in daily practice and an expectation of benefit is likely to promote benefits associated with more standard forms of MT. For this reason, the present study is purposefully agnostic as to neurofeedback algorithm, but instead investigates whether commercial N-tsMT promotes benefits relative to an active control, non-meditative training condition.

Here, we present the first empirical investigation of the effectiveness of a commercial N-tsMT system to assist participants in a self-guided, 6-week home-based practice. Relative to a randomized, active-control training condition, participants were assessed on our hypothesized target measures of attention and well-being before and after training. The goal of the study was to investigate whether N-tsMT could benefit attention and/or well-being in an ecologically-valid research paradigm. Specifically, we hypothesized that 6 weeks of N-tsMT would promote greater improvements to well-being and attention relative to training in the active control condition.

## Methods

We compared 10 min of daily N-tsMT against a cognitively-demanding active control training condition in healthy adults over a 6 week period. Participants were randomly assigned to condition with equal allocation to each condition. At baseline and following training, participants were assessed on a variety of attention and affective measures, in addition to completing daily assessments of mood, stress, and practice quality throughout the training period. Concurrent research-grade EEG was also acquired during baseline and post-intervention testing, and will be described in a subsequent report.

### A priori power analysis

The current study was designed to efficiently test for the types of effects commonly observed in conventional, group based MT interventions. In our prior work, between-groups effects on depression symptoms in an MT group vs. waitlisted controls were very large, with effects greater than d = 1.3 [34]. In other work, effects on attention as measured by the Stroop task were again large, with d = 1.1 [25]. We planned a mixed-model design here to improve efficiency, targeting the interaction between experimental and control groups and within time (pre and post intervention). Using the G*Power application [46], we estimated the required sample size to detect large within-between interaction effects with 90% power. Assuming that some of our previously observed effects were due to uncontrolled expectancy in our waitlist designs, we employed a more conservative estimate of a large effect size, *d =* .6/*f* = .3, combined with a previously observed [34] correlation among repeated measures of *r =* .66, with 2 comparison groups and 2 measures. The analysis suggested that a total sample size of *N* = 22 would be sufficient for the analysis; estimating some dropout from each group, we planned to collect a total *N* = 30 for the present study.

### Participants

Healthy, community dwelling, adult participants were recruited between January 2015 and May 2015 from an online participant database at the Rotman Research Institute at Baycrest Health Centre in Toronto, Canada, as well as through online advertisements posted to Craigslist, an online classified ad site. All participants were required self-identify as being healthy but under moderate to high levels of stress, to be fluent in English and have normal or corrected to normal vision. Participants were also required to have daily internet access for the purposes of completing daily training and experience sampling. Exclusion criteria included the presence of any neuropsychological or psychiatric condition that may influence the functioning of the nervous system, a history of head injury, or prior meditation experience. Recruitment completed when 15 participants in each group (*N* = 30) had successfully completed training and attended the post-intervention assessment.

While the use of mindfulness techniques seems promising for particular mental disorders, the current study was aimed at high functioning, community dwelling adults who are most likely to be early adopters of this technology. Furthermore, it should be noted that the most popular mindfulness interventions (Mindfulness Based Stress Reduction – MBSR; and Mindfulness Based Cognitive Therapy- MBCT) are not currently indicated for major psychiatric disorders- MBSR is commonly offered to community dwelling adults dealing with elevated levels of stress [47], and MBCT to people currently remitted from depression but who may be at risk for relapse [48]. Thus in keeping with the literature that supports MBSR and MBCT efficacy, we sought to first test N-tsMT on the most general and safest sample of participant, i.e., healthy, community dwelling adults, who nonetheless self-identify as carrying a moderate stress burden. Psychiatric disorders were likewise ruled out through self-report, i.e., participants had to endorse that they were healthy without any major medical or psychiatric conditions as part of the intake interview during recruitment to the study.

Randomization was performed using the random number generator function in the MATLAB programming environment [49], which was used to randomize sub-blocks of 4 participants equally to the experimental and active control conditions. Randomization was conducted by the principal investigator (NF) and communicated to research assistants without any participant contact. Initial randomization successfully matched age and gender across experimental groups. Participants were subsequently withdrawn from the study if they either expressed a desire to cease participation, or failed to meet practice adherence criteria of at least 75% daily practice over the course of the study, and no fewer than two practice sessions per week. Withdrawal rates for the two groups were not significantly different. Following study completion, participants were also withdrawn from final analysis if their performance on the primary behavioral attention task was below 50% accuracy, as mean performance on the task even before such exclusion was 86.5%.

**Fig. 1:**
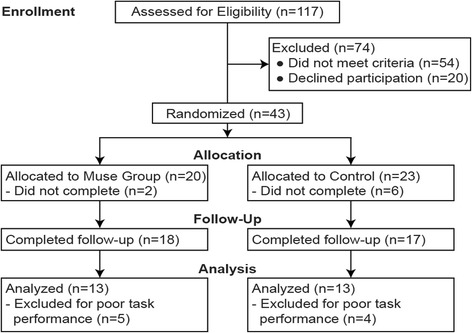
CONSORT diagram of the study participants

The study adhered to all CONSORT guidelines. There were no gender or age-related differences between groups at any point during the study, and Chi-square analyses of participant dropout showed no differences in gender or age. The CONSORT diagram for the study is presented in Fig. 1. The final sample included in the study consisted of 13N-tsMT participants (seven Males, mean age 33.3, SD = 4.7) and 13 Control group participants (seven Males, mean age 32.0, SD = 4.9). All participants were included in all data analyses.

### Materials

Participants completed both laboratory assessment at baseline and post-intervention, as well as daily experience sampling questionnaires after each training period. During laboratory assessment, participants completed primary measures of attention and well-being, as well as a short battery of exploratory measures to examine the transfer of hypothesized training effects. The complete study dataset is available in de-identified form online as an Additional file 1 entitled “Complete Study Data”.

#### Neurofeedback

To deliver the N-tsMT intervention, we employed Interaxon Inc.’s Muse (RRID:SCR_014418)﻿, a wireless EEG headset and accompanying mobile device software application. The headset has four dry sensors (two mastoid and two forehead sensors) and fits over the ears and extends at an angle over the middle of the forehead when properly fitted. Data were sampled at 220 Hz and referenced to the Fpz channel. Data were communicated wirelessly to the mobile device application.

To provide high-fidelity neurofeedback, the Muse algorithm promoted a proprietary combination of frequency bands that the company describes as having been associated with meditative states, e.g., [50]. In addition, the software application provided a guided pre-session calibration to customize neurofeedback to match participant experience prior to each training session. Calibration involved two brief exercises: in the first exercise, participants were asked to perform a word association task to simulate a period of mind-wandering. In the second exercise, participants were asked to relax and clear their minds as a brief induction of a focused attention state. These two calibration conditions were then entered into a machine learning algorithm to generate a session-specific signature of concentration and distraction customized to the participant. Calibration lasted 1 min. Following calibration, guided meditation instructions were delivered through the paired iPod, directing attention towards breath sensation. Neurofeedback was delivered through auditory cues of wind and storm sounds, which increased in intensity with greater estimated distraction, and subsided towards calm with greater estimated stability of attention.

#### Primary measures

The primary measure of attention selected was the Stroop task, a classic test of attention and executive function [51, 52], which has shown sensitivity to meditation experience in the research literature [53]. In the Stroop task, stimuli were presented one at time from the set of words “BLUE”, “RED”, “GREEN” or “YELLOW”, with each word coloured blue, red, green, or yellow. The participant’s task was to respond to the colour of the word by pressing one of four keyboard keys mapped to the colours: blue, red, green, and yellow. Participants completed a practice session to memorize the key mappings with the colours. In congruent trials, the word matches the colour of the word. In incongruent trials, the word does not match the colour of the word and thus interferes with the participant’s response to the colour, resulting in slower responses. The effect of interference was measured as the difference in response times between incongruent trials and congruent trials for correct trials. Each trial began with a fixation cross for 500 ms, followed by the stimulus word for 200 ms, a response window of 1000 ms, and an inter-trial interval of 1000 ms. Participants completed a total of 480 trials divided across ten blocks. Each block consists of 32 congruent trials and 16 incongruent trials.

The primary measure of affect was the Brief Symptom Inventory (BSI), a well-validated and popular self-report measure of psychological distress [54–56]. The BSI taps into three major domains of affective health, namely depression, anxiety, and somatic symptoms, the three major areas in which meditation interventions show the most reliable and pronounced therapeutic efficacy [57]. The BSI consists of 18 items and shows good internal validity and reliability across a variety of cultures and clinical populations [58–60]. The BSI was delivered through an online questionnaire portal using Qualtrics software (Qualtrics, Provo, UT).

#### Exploratory measures

At baseline and post-intervention laboratory testing, participants completed a short online battery of questionnaires intended to measure transfer of training benefits to related domains of attention and affective processing. Testing was completed in a quiet behavioural testing room with a trained research assistant. The cognitive tests and questionnaires took approximately 40 min to complete.

In the domain of attention, participants completed the d2 and digit span tasks. The d2 task is a test of concentrative attention that provides a reliable and internally valid index of visual scanning accuracy and speed [61]. In the task, participants were asked to scan a row of characters and cross of any letter “d” with two marks above, below or one on either side. Stimuli were presented with distractors similar to the target, such as letter “p” and fewer or more than two marks. Participants had 15 s to complete each row, after every 15-s interval, they moved onto the next row for a total of 15 rows. In the event that participants completed a row early, they were asked to wait until the interval was over before moving to the next row. The task produces participant scores for errors of commission and omission in detecting the target stimuli.

The digit span task is a measure of working memory that may be impacted by changes to attentional control [62]. In the task, participants were asked to repeat a list of digits in the same order as was said to them (forward digit span), each list consisted of eight set of numbers. The lists are progressively harder, as an extra digit gets added to the successive lists. Testing ceased if participants made errors on more than two sets of numbers; the list at which the participant successfully repeated 5 of 6 sets of numbers correctly was the participant’s forward span. A similar metric was applied for backwards span, in which participants are asked to repeat back sets of numbers in reverse order. Testing ceased when participants made two incorrect responses, and the participant’s backward digit span was the list in which they got at least 2 out of 3 sets of numbers correct.

In the affective domain, a series of well-validated psychometric instruments were employed. To gauge levels of dispositional mindfulness that may have been sensitive and/or predictive of the training intervention, participants completed the Freiburg Mindfulness Inventory (FMI) [63]. To measure current emotional state participants completed the positive and negative affective schedule (PANAS) to assess mood at the time of testing [64]. To assess the generalization of physical and affective symptoms to broader appraisals mental and physical health, participants completed the brief version of the World Health Organization Quality of Life scale (WHOQOL-BREF) which measures domains of overall well-being, as well as subscales for physical, psychological, social, and environmental well-being [65]. Lastly, participants also completed the Big Five Inventory (BFI) personality checklist, to examine whether practice could shift such dispositional variables, and also to explore whether personality traits might predict intervention responsiveness.

#### Daily experience sampling

Following each practice session, participants were asked to complete a brief online survey. The survey employed a 7-item Likert format, with questions designed to gauge daily fluctuations in user experience in the domains of emotional valence (“pleasantness”), arousal (“emotional activity”), ability to focus, quality of the instruction/feedback, perceived effort, calmness, body awareness, and stress (specific question wording is available as an Additional file 2 online entitled “Daily Experience Sampling Items”). At the end of each report, participants also had the opportunity to communicate technical difficulties or give other comments. The questions were accessed through an online survey website (Qualtrics, 2015; Provo, Utah, USA) wherein participants identified themselves via a unique ID number.

### Procedure

Following initial telephone screening interviews, participants were invited to attend assessment sessions at the Rotman Research Institute at Baycrest Health Centre in Toronto, Canada. Participants completed a short battery of attention and executive control tasks, and self-report measures of well-being. Participants were blind to experimental condition while completing the baseline assessment battery, before being informed of their group assignment to the N-tsMT (Muse) or active control (Khan Academy Math) conditions. Participants were trained on their respective intervention conditions.

#### N-tsMT

Participants were provided with a Muse headset, iPod with the pre-installed Calm App, charging cables and headphones. Participants were taught to set up the Muse headset and associated software application, which delivers a guided-meditation application focusing attention on the breath, a core introductory meditation practice in MT [47]. The application provided step-by-step instructions on operating the headset and guided participants through N-tsMT sessions.

After fitting the headset, the quality of the recording was indicated by a coloured connectivity bar in the meditation software. If the connectivity bar was not full, the user would check to see if the sensors are clean and adjust the positioning of the headset to ensure sensors had good skin contact. Users began each mediation session by clicking on an icon that prompted voice-recorded guided meditation. During the meditation, the Muse headset collected data and transmitted the information to the application, which provided real-time auditory feedback during the meditation session, such as beach waves and wind sounds that grew louder and more intense if increasing mind-wandering was detected. A calm score was calculated at the end of each session, which reflects the percentage degree of focused attention detected during the session. At the end of the training session, participants completed a daily internet survey to report on their experience via a unique ID number.

#### Khan Academy math training

Participants were enrolled in a free, online, high school level algebra class, in which they were presented with a mixture of brief lectures and math problems. Daily training consisted of completing 10 min of course material. The program allowed participants to learn concepts through feedback/hints, and watching videos demonstrating how to solve similar problems. At the end of each concept learned, participants received a score of correct responses and awarded a mastery level to move on to the next concept. At the end of the training session, participants completed a daily internet survey to report on their experience via a unique ID number.

#### Expectancy

To control for expectancy, participants in both conditions were told the purpose of the study was to compare the effects of different types of technology-supported training rather than framing the study around mindfulness meditation. Participants were informed that daily mental exercise has the potential to improve attention and well-being, even if it is effortful or boring to perform the practice itself. No participant communicated disbelief with this claim, even after being assigned to their experimental condition. The framing we employed was deliberate in order to reduce differential expectancy or desirability bias between the groups.

#### Daily training

The daily training lasted 6 weeks (42 days). Participants were required to complete at least 32/42 (75%) sessions over the 6 weeks of training. A successful training session consisted of completing either a 10-min meditation session with the Muse or completing 10 min of algebra practice problems on Khan Academy. Individuals also completed a short daily survey to report their engagement and satisfaction levels with the current practice. Daily practice data from the EEG headsets was automatically uploaded to an encrypted server. Daily practice data for Khan Academy was accessible through the coach account. The daily questionnaires, daily practice EEG data and daily reports from Khan Academy were used as a measure of adherence and performance of the daily practices. Completion of the daily sessions was monitored through daily survey completion reports and server reports. Individuals who missed two consecutive sessions were sent an email or phone reminder to ensure adherence.

#### Compensation

Participants received compensation for the two lab sessions as well as the daily sessions prorated to the number of session they completed. Transportation costs were also covered and a bonus incentive of $20 was included for participants who completed 75% or more daily training sessions.

### Analysis

Given the small sample size in this study, we guarded against violations to normality by employing non-parametric analyses using the R statistical computing environment [66]. For all variable of interest, Wilcoxon Rank Sum tests were used to investigate within-participant training effects. Post-training – pre-training difference scores were computed as an estimate of training effects. These scores were then compared between groups in a further Wilcoxon Rank Sum test, equivalent to parametric Time X Group interactions. It should be noted running mixed model (Time x Group) ANOVAs, which assume normality of distributions, did not alter the pattern of findings described below.

- Attention. Attention was measured by assessing reaction time (RT) on the Stroop task, using correct trials only. Two measures were evaluated: average RT across both congruent and incongruent trial conditions as a measure of attention speed, and the incongruent – congruent RT costs scores as a measure of conflict resolution.
- Well-Being. The three BSI subscores (somatic, depression, and anxiety symptoms) were separately evaluated.
- Attention/Affect association. Relationship between primary measures of attention (Stroop) and symptom (BSI) changes were assessed through bootstrapped regression using the Bootstrap Function package (“boot”) [67, 68] the R statistical computing environment [66]. Bootstrapped regression is similar to conventional linear regression but also examines subsets of the participants to minimize the influence of outliers. No differences in the significance of associations were observed using bootstrapped as opposed to traditional linear regression.
- Dispositional predictors of treatment response. Several exploratory bootstrapped regression analyses were computed using the Bootstrap Function package (“boot”) [67, 68] the R statistical computing environment [66] to examine the relationship between baseline dispositional mindfulness (FMI) and personality (BFI) and changes in the primary measures of attention and well-being that were sensitive to the N-tsMT intervention. This analysis was applied to the N-tsMT group only as an a priori sample of interest.
- Daily experience sampling. Experience sampling variables were subjected to growth curve analysis in the R statistical programming environment [66], using the non-linear mixed effects package (“nlme”) [69] to examine changes related to daily practice. The modelling employed a Restricted Maximum Likelihood Estimation (REML) method to model the effects of group, time, and the group x time interaction. Intercepts were set to random to allow for individual differences in the effects of these variables. Model comparison between fixed and random slopes revealed no improvement in model fit for letting slopes vary across individuals, so fixed slopes models are included in the current report. A similar evaluation of including an autoregressor function (AR1) to control for association between temporally proximal measurements revealed no improvement in model fit, and was therefore excluded from the reported model.
- Correction for multiple comparisons. To be considered significant, a priori analyses were Bonferroni corrected for multiple comparisons across the evaluation of the primary measures. Exploratory analyses were not corrected for multiple comparisons, and are presented for their descriptive rather than inferential value.

## Results

### Attention

**Fig. 2:**
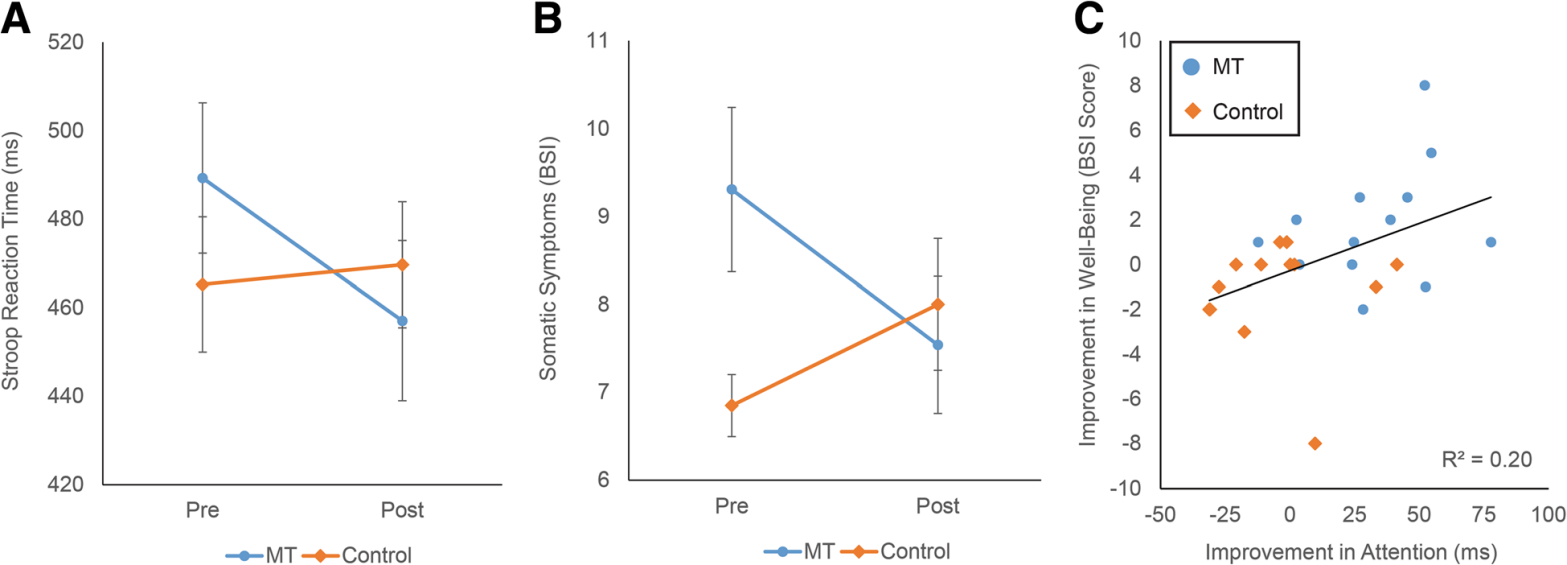
Training effects on primary measures of Attention and Well-Being. Panel **a** Time x Group interaction on Attention, as indexed by Stroop task mean RT. Panel **b** Time x Group interaction on Well-Being, for BSI Somatic Symptom scores. Panel **c** Relationship between training-related changes in Attention and Well-Being. Reductions in Stroop RT and BSI Somatic Symptoms are both displayed as positive values, i.e., greater scores demonstrate greater reductions. Interactions in Panels **a** and **b** are significant at *p* < .05, corrected for multiple comparisons among primary measures. Error bars are standard errors

Analysis of overall Stroop RT revealed a significant interaction between group and time, Z = 3.29, *p* < .001, *r =* .65, such that N-tsMT uniquely improved processing speed, despite equivalent accuracy between groups and time points (Table 1; Fig. 2a).

**Table 1:** Summary of training effects

	MT			Control			
	Baseline	Post-intervention	Change	Baseline	Post-intervention	Change	Time x Group r
Primary measures							
*Attention (Stroop)*							
Mean RT	489.3 (61.3)	457.0 (65.4)	**−32.3 (15.4, 49.0)**	465.2 (55.1)	469.7 (51.4)	4.4 (−17.6, 9.7)	**.65**
Interference Cost	129.5 (56.0)	98.1 (37.9)	**−31.4 (6.0, 57.7)**	97.0 (35.4)	88.1 (33.9)	−8.9 (−13.4, 30.5)	.27
*Well-Being (BSI)*							
Somatic	9.3 (3.4)	7.5 (2.8)	**−1.8 (−4.0, −0.0)**	6.8 (1.3)	8.0 (2.7)	1.2 (0.0, 5.0)	**.55**
Depression	9.7 (4.7)	8.9 (3.4)	−0.8 (−1.5, 3.5)	8.8 (3.8)	8.8 (4.3)	0.1 (−2.0, 1.5)	.15
Anxiety	6.4 (3.0)	5.2 (1.4)	−1.2 (−0.5, 2.5)	5.9 (2.5)	5.8 (2.6)	−0.1 (−2.0, 2.0)	.22
Exploratory measures							
*Attention*							
Digit span							
Forward span	6.2 (0.8)	5.9 (1.2)	−0.3 (−1.0, 1.5)	6.3 (1.1)	6.6 (1.0)	0.3 (−1.0, 0.0)	.31
Backward span	4.7 (1.4)	4.5 (1.0)	−0.2 (−1.0, 1.0)	5.8 (0.9)	5.2 (1.3)	−0.6 (−0.5, 2.0)	.19
D2 Test							
Commit Error %	0.5 (0.7)	0.3 (0.4)	−0.2 (−0.1, 0.5)	0.9 (1.8)	0.9 (2.0)	0 (−1.5, 1.6)	.20
Omit Error %	9.0 (5.4)	7.4 (5.6)	**−1.6 (−3.0, −0.1)**	9.1 (6.2)	8.4 (5.5)	−0.7 (−0.6, 2.2)	.27
*Well-Being*							
Mindfulness	38.2 (7.4)	37.3 (9.0)	−0.8 (−3.0, 4.5)	39.8 (3.8)	39.9 (4.8)	0.2 (−4.5, 4.0)	.08
Positive affect	32.8 (4.8)	34.3 (6.5)	1.5 (−4.5, 1.0)	34.4 (5.0)	34.6 (4.4)	0.2 (−2.5, 1.5)	.18
Negative affect	22.8 (7.2)	18.2 (5.2)	**−4.7 (−1.0, −10.0)**	21.5 (5.9)	20.4 (7.2)	−1.1 (−0.5, 3.5)	.26
Quality of life							
Overall	4.2 (0.8)	4.0 (0.7)	−0.2 (a)	3.9 (0.6)	3.9 (0.5)	0.0 (−1.0, 1.0)	.14
Physical	26.6 (4.7)	27.2 (4.7)	0.5 (−2.5, 1.0)	28.2 (2.6)	28.8 (3.0)	0.5 (−3.0, 2.0)	.03
Psychological	21.2 (3.3)	22.2 (2.8)	**1.1 (−2.5, −0.0)**	22.0 (2.8)	21.6 (2.8)	−0.4 (−1.5, 2.0)	.36
Social	10.8 (2.0)	11.8 (2.0)	0.9 (−3.0, 0.5)	11.2 (2.0)	11.2 (2.2)	0 (−1.5, 1.5)	.24
Personality							
Extraversion	26.4 (6)	26.4 (5.9)	0.0 (−2.0, 2.0)	26.7 (4.9)	27.2 (5.0)	0.5 (−2.0, 1.0)	.20
Agreeableness	35.5 (5.5)	35.2 (5.8)	−0.2 (−2.5, 3.0)	34.7 (7.5)	34.0 (6.9)	−0.7 (−0.5, 2.0)	.06
Conscientiousness	29.8 (6.7)	30.8 (5.2)	0.9 (−3.5, 2.0)	31.7 (6.5)	31.1 (6.1)	−0.6 (−1.0, 3.0)	.33
Neuroticism	20.5 (8.2)	19.5 (6.6)	−1.1 (−1.0, 3.5)	20.4 (4.8)	20.8 (4.7)	0.5 (−3.0, 2.0)	.25
Openness	39.3 (7.8)	40.2 (7.2)	0.8 (−4.0, 2.0)	38.2 (4.5)	38.0 (4.7)	−0.2 (−2.0, 3.0)	.09

The mean scores of each measure are displayed for each training group are displayed with standard deviations in parentheses. Mean within-group change scores are displayed with 95% confidence intervals computed from non-parametric tests. Effect sizes (r) for the group x time interaction are displayed in the rightmost column. For primary measures, effects that are significant at *p* < .05, *corrected* for multiple comparisons are in bold. Exploratory measure effects that are significant at an *uncorrected p* < .05 are displayed in bold

^a^Observation ranks were tied; no non-parametric confidence interval was available

Our a priori hypothesis predicted changes to Stroop interference costs, rather than overall RT. While the N-tsMT group showed a numerically greater reduction in interference costs than the Control group (31 ms vs. 9 ms), the interaction between group and training was not significant, Z = 1.38, *p* = .17, *r =* .27. No training effects were observed for Stroop task accuracy. Exploratory measures of attention, such as the digit span and d2 tests, did not reveal any training effects.

### Well-being

A significant interaction was observed between group and time on the Somatic Symptom subscale of the BSI, Z = 2.81, *p* = .004, *r =* .55, such that N-TSMT significantly reduced somatic symptoms relative to the Control group. No effects were observed for the depression or anxiety factors of the BSI, nor for the exploratory measures of mood, mindfulness, and quality of life.

### Attention/Well-being relationship

Improvements in somatic symptoms were predicted by changes in Stroop RT, r(24) = .44, *p* = .024, such that greater improvements in RT predicted greater reductions in somatic symptoms (Fig. 2, Panel c). This association was not apparent between Stroop RT and depression and anxiety subscale scores of the BSI.

### Dispositional predictors of treatment response

Of the dispositional indicators at baseline, only neuroticism was related to training-related changes in the N-TSMT group. Somewhat surprisingly, *higher* neuroticism was associated with greater reductions in somatic symptoms, r(11) = −.70, *p* = .007. This relationship was not observed within the Control group, r(11) = .18, *n.s.* Changes in Stroop performance were not associated with baseline dispositional variables.

### Experience sampling

**Fig. 3:**
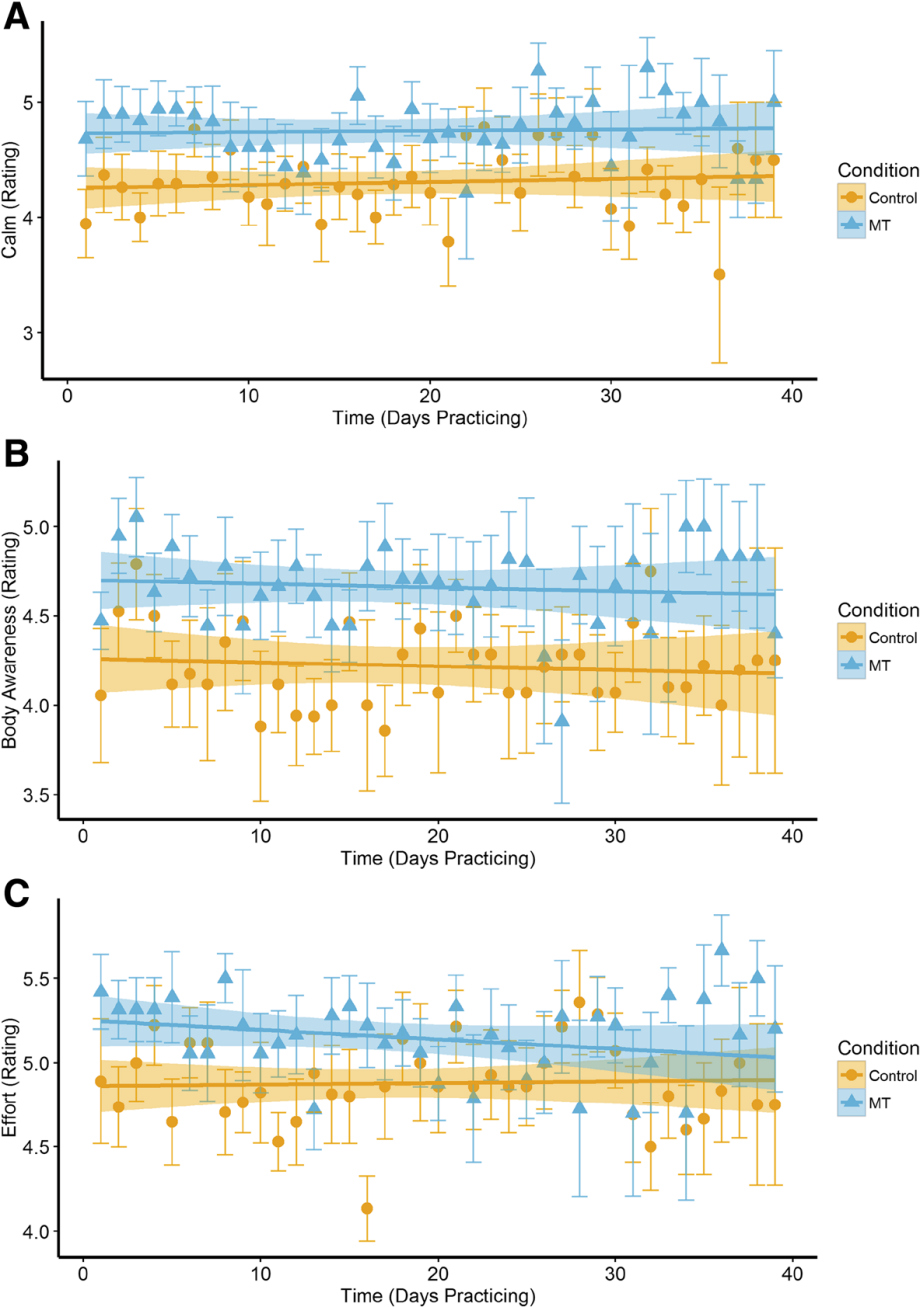
Daily experience sampling effects. Plots are generated using the beta values from the growth curve model Group x Time using data obtained over the training period. Effects of Group and Time on self-reported feelings of: **a** Calm, **b** Body Awareness, and **c** Effort. Main effects are significant at *p* < .05, uncorrected, as is the Time x Group interaction in Panel **c** (Effort). Error bars are standard errors

Participants averaged 32 (SD = 9.2) daily responses over the 42 day training period (Table 2). Both groups were equally adherent to training. The N-TSMT group consistently reported greater calm following practice sessions than the control group, t(36) = 2.16, *p* = .04 (Fig. 3, Panel a), and greater body awareness, t(36) = 2.03, *p* < .05 (Panel b). The N-TSMT group reported putting in significantly greater effort than the control group, t(36) = 2.54, *p* = .02, an effect that reduced over time, as expressed through a group x time interaction, t(1047) = −2.00, *p* = .046 (Panel c). No effects on daily stress or the other daily experience measures were observed. Average experience sampling variable scores were not significantly correlated with changes in attention and well-being. Baseline effort was included as a potential moderator of the attention and well-being models, but did not interact significantly with Group and Time to predict our primary dependent variables. Dispositional neuroticism, which was positively linked to treatment response, was included as a post hoc moderator in the experience sampling models for calm, body awareness, and effort, but did not contribute significantly to any of these models.

**Table 2:** Summary of experience sampling growth curve effects

	Intercept	Time	Group	Time x Group
Body	**4.23**	0.00	**0.50**	0.00
Calm	**4.25**	0.00	**0.51**	0.00
Emotional activity	**4.36**	0.00	−0.30	0.00
Feedback quality	**4.52**	0.00	−0.05	0.01
Focus	**4.68**	0.00	−0.16	0.01
Pleasantness	**4.54**	0.00	0.27	0.01
Effort	**4.86**	0.00	**0.47**	**−0.01**
Stress	**3.47**	0.01	0.33	−0.01

Beta weights for each experience sampling variable are displayed. Beta values that are significant at an uncorrected *p* < .05 significant threshold are displayed in bold. Marginal effects, i.e. .05 < *p* < .1, are displayed in italics. For group, N-tsMT is coded as 1 and Control as 0

## Discussion

This is the first experimental study to examine the benefits of N-tsMT in a healthy community sample relative to an active control group. We investigated the consequences of 6 weeks of daily 10 min training sessions, contrasting breath-focused meditation against algebra exercises. The study assessed training effects on two primary dependent variables: attention and well-being. We hypothesized that training would benefit both attention and well-being, and these hypotheses were partially supported. Numerous additional exploratory variables were also included to establish the specificity of the training effects- our findings give no indication that the training transferred to these exploratory domains.

The primary measure of attention was the Stroop task, one of the most widely studied behavioural measures of attentional control. The primary dependent variable associated with Stroop performance is the interference score, the RT cost of naming color/name incongruent words relative to color/name congruent words. However, the Stroop task also affords a measure of overall attention speed in the form of average RT across the task. Relative to active control, N-TSMT was uniquely associated with faster RT across the Stroop task, an effect apparent in both the congruent and incongruent conditions. It may therefore be reasonable to infer from our data that the N-tsMT intervention enhanced attention speed, but did not specifically affect interference resolution. The observed effect size for Stroop interference in this study (*r =* .27) is equivalent to a Cohen’s d = .56, which is much smaller than the d = 1.1 reported in the literature [25]. This difference may stem from a weaker effect of N-tsMT compared to more intensive meditation, or from the current study’s use of an actively-controlled, pre-post interaction design as compared to prior reports of cross-sectional designs. The enhanced attention speed effect amounts to a d = 1.7, an effect not previously reported in literature and requiring further investigation.

The primary measure of well-being was the BSI, a well-validated clinical instrument for detecting and monitoring symptoms of depression, anxiety and somatization. We hypothesized meditation-unique improvements across all domains on this measure. The data partially supported this hypothesis, showing reductions in somatic symptoms following N-tsMT relative to active control. The effect size for this change in somatic symptoms (*r =* .55) is equivalent to a Cohen’s d = 1.3, which mirrors affective symptom change in our prior research [34]. However, these prior studies showed effects across a variety of affective symptom domains; in the current study, depression and anxiety symptoms were not significantly affected by training. Indeed, the effect sizes for reductions in depression (d = .3) and anxiety (d = .45) were much smaller than those found in the literature. Given the focus of the MT instructions on the somatic experience of the breath, it is perhaps unsurprising that training effects were limited to the somatic domain, but it suggests that greater levels of practice, or the introduction of other components of manualized interventions are required to promote more general improvements across affective symptom domains. At the least, we may infer that the N-tsMT intervention has promise for improving healthy participants’ relationships with somatic experience, reducing negative appraisals of the varied physical sensations of daily life.

An intriguing relationship was observed between training-related changes in attention and well-being, such that improvements on Stroop RT were moderately associated with reductions in somatic symptoms. This relationship was largely maintained within the N-tsMT group, belying suggestions that this observed correlation is simply an artifact of correlating two variables selected on the basis of their group by time interactions. The causal relationship between changes to attention and subjective well-being cannot be inferred in the current study design, and it seems equally plausible that better attention allows for greater feelings of well-being, but conversely reduced intrusive thoughts around somatic symptoms might also free attentional resources and improve performance. However, as the N-tsMT instruction focused on the stabilization of attention, it seems that attention changes may be more proximal markers of the training. Furthermore, the coupling between improvements in attention and well-being is consistent with a contemplative training literature that proposes the stabilization of attention as a resource for “skillful means”, the ability to clearly and directly care for oneself and others even in the face of acute stress. Nevertheless, the hypothesis that attention changes *cause* improvements in well-being requires further investigation.

Of the baseline indicators included in the study, only neuroticism predicted training-related changes. This prediction was limited to somatic symptoms and not Stroop performance, consistent with the well-established literature associating emotional volatility with reports of affective distress. This finding suggests that the therapeutic benefits of N-tsMT may be particularly evident in individuals with chronic tendencies towards worry and emotional volatility, avoiding symptom ‘floor effects’ that may be evident in an asymptomatic healthy community sample.

To better appreciate the quality of participant experience during training sessions, a daily experience sampling approach was also employed. Relative to active control, N-tsMT provoked greater feelings of calm and body awareness following each training session. N-tsMT was initially perceived as more challenging than the active control condition, although reported meditation effort declined with time, until it was indistinguishable from control condition effort by the end of the 6 week period.

Together, the data support the idea that repeated brief sessions of N-tsMT improve attention and well-being relative to an active control math learning condition. However, the effects of training are specific, with improvements limited to faster overall RT and reduced somatic symptoms. Related measures of attention, and related indices of well-being such as depression and anxiety symptoms, quality of life, or dispositional mindfulness were not specifically enhanced by N-tsMT. This lack of transfer to related domains suggests that greater practice time, more general MT instructions, or some combination thereof might be required to replicate the broader benefits of more intensive, group-led meditation programs.

### Limitations

One clear limitation to the present exploratory study lies in its sample size. While 13 participants per group is relatively common for training intervention studies, it still warrants replication in larger samples would to firmly validate these promising effects. Our power analyses were designed to detect medium-to-large effects, and so we must remain agnostic to the possibility that a large sample would have detected more modest effects in the non-significant measures. Nonetheless, participants retained in the study were well-matched between the groups, and showed equivalent practice adherence. Given such tight controls, it is encouraging that positive effects of training were observed in our primary outcome measures, as an actively-controlled mixed-model intervention design yields a comparable quantity of measurements to a cross-sectional study twice its size.

A second limitation to this study is that it cannot adjudicate between competing mechanistic accounts of the study findings. Traditional MT interventions are likely efficacious due to multifaceted blends of meditation practice, didactic content, group support and positive expectancy effects [70]. The current N-tsMT study removed the element of group support and didactic content but introduced neurofeedback, and so it is impossible to distinguish whether study findings are a consequence of the neurofeedback, positive expectancy, or meditation practice itself. However, the goal of the study was not to dismantle N-tsMT to identify a specific mechanism, but rather to gauge whether N-tsMT promoted benefits comparable to a literature of in-person, facilitator-led, group interventions. Without documented evidence of efficacy, there is little point to searching for a specific mechanism. Thus we explicitly make no claim that the neurofeedback was the active element in producing the study effects, but rather remain agnostic as to the underlying mechanism.

A third potential issue with the current study is that we provided multiple incentives for daily practice, both in the form of pro-rated compensation per day of practice, and in the form of e-mail and phone reminders should participants miss subsequent days of practice. Such incentives may have elevated our adherence levels relative to more naturalistic usage of the training technology. However, adherence was not significantly lower for the N-tsMT condition relative to active control, suggesting that despite greater effort reported in completing earlier N-tsMT sessions, the training is comparably engaging compared to online training courses. As such, adherence issues are not likely to be of greater concern for N-tsMT than for other electronic learning technologies.

A more general limiting implication to this research lies in the real-world significance of the observed effects. We did not observe transfer of benefits across tests of attention beyond our primary measure, and even our primary measure of well-being only truly showed effects in the domain of somatic symptoms. The limited effects on well-being are perhaps due to the focus of the MT to concentrate attention on visceral sensation of the breath rather than broader mood state or social factors. Thus, to generalize benefits, future implementations of N-tsMT may require practices that tap into broader affective domains, analogous to the progression of content in standardized 8-week group meditation courses such as MBSR or MBCT. Another clue around transfer comes from the somewhat surprising finding that participants with the *highest* baseline neuroticism scores were the ones to benefit the most. It may be that our ability to observe clinically-meaningful changes is limited in a relatively healthy community sample- larger effect sizes may be apparent in clinical populations who begin treatment with elevated markers of anxiety, depression, and generalized stress.

In conventional MT programs, meditation practice begins with body sensation but progresses more broadly to include awareness of sounds, feelings, and thoughts. Such programs also include informal practices aimed to facilitate the use of mindful attention in daily life. It seems that with greater breadth of practices, transfer of beneficial effects across affective domains are more likely to be observed. Furthermore, such programs assign over 40 h of formal meditation practice over an 8-week period, whereas here we requested only 7 h total practice for full adherence. Issues of minimal dose and meditation style are the topic of ongoing investigation in the contemplative literature and will hopefully be clarified with further study.

## Conclusions

Here we have presented the first actively-controlled evidence that N-tsMT delivered through consumer-grade EEG promotes promising benefits for attention and well-being. While our findings are modest, they provide a supportive indication for the feasibility and utility of such technology for client-centered training for positive self-transformation. How best to further validate, optimize and generalize the effects of such training will be of considerable interest as the marketplace for such adaptive technologies matures.

## Additional files

Additional file 1: Complete Study Data. Complete study data from the baseline and follow-up assessments and the daily experience sampling data. (CSV 32 kb)
Additional file 2: Daily Experience Sampling Items. A listing of the daily experience sampling questionnaire items used in the study. (PDF 142 kb)

## Abbreviations

BSI: Brief symptom inventory
FMI: Frieberg mindfulness inventory
MT: Meditation training
N-tsMT: Neurofeedback-assisted technology-supported meditation training
PANAS: Positive and negative affect schedule
REML: Restricted maximum likelihood estimation
RT: Reaction time
tsMT: Technology-supported meditation training
WHOQOL-BREF: Brief Version of the World Health Organization quality of life scale
