# Supplementary material for: Attentional and affective consequences of technology supported mindfulness training: a randomised, active control, efficacy trial
Source: BMC Psychol. 2016 Nov 29;4:60. doi: 10.1186/s40359-016-0168-6 (PMC5127005; doi:10.1186/s40359-016-0168-6)
Supplement: Additional file 2: — Daily Experience Sampling Items. A listing of the daily experience sampling questionnaire items used in the study. (PDF 142 kb) [file 40359_2016_168_MOESM2_ESM.pdf]

Daily Experience Sampling Items

1. *Pleasantness*. I feel ________ after completing today's session. (Very Unpleasant / Very Pleasant)
2. *Emotional Activity*. I feel ________ after completing today's session. (Inactive / Very Active)
3. *Focus*. I was ________ during today's session. (Very Distracted / Very Focused)
4. *Feedback.*

   MT: I felt the wind feedback was ________ of mind wandering during today's session. (Very Unreflective / Very Reflective)

   Control: I felt the level of instruction was ________ during today's session. (Very Unhelpful / Very Helpful)
5. *Effort*. I put in ________ effort during today's session. (A Little / Very Much)
6. *Calmness*. I feel ________ now than I did before today's session. (Less Calm / More Calm)
7. *Body Awareness*. I feel _______ to my body right now. (Very Disconnected / Very Connected)
8. *Stress*. Today was _______ stressful. (Not at All / Extremely)
